# Predictive value of serum d-serine level for hearing impairment in uremic patients

**DOI:** 10.1080/0886022X.2023.2194448

**Published:** 2023-04-03

**Authors:** Jiaqing Li, Dunlu Yuan, Qing Yang, Jingjing Huang, Zhu Zhou, Ruomei Li, Qing Li

**Affiliations:** a Department of Nephrology, The First Affiliated Hospital of Kunming Medical University, Kunming, China; b Department of Medical Record, The Third People’s Hospital of Kunming, Kunming, China; c Department of Otolaryngology, The First Affiliated Hospital of Kunming Medical University, Kunming, China

**Keywords:** d-Serine, uremic, hearing impairment, predictive value

## Abstract

**Objective:**

To investigate the predictive value of serum d-serine level for hearing impairment (HI) in uremic patients.

**Methods:**

In this study, 30 uremic patients with HI and 30 with normal hearing were selected. The basic conditions, biochemical indicators, and serum serine levels of the two groups were compared to analyze the influencing factors of HI.

**Results:**

The age and d-serine levels were higher in the HI group, while the l-serine level was lower than uremia in the normal hearing group. Logistic regression analysis showed that d-serine level ≥10 μM and older age increased the risk of HI. The area of the receiver operating characteristic (ROC) curve drawn by the prediction probability of HI was 0.838, indicating that age, d-serine, and l-serine had predictive diagnostic values for HI (*p* < .001). Among these, the ROC curve area of d-serine in predicting HI in uremic patients was 0.822 (*p* < .001).

**Conclusions:**

Increased d-serine and age are two risk factors for HI, while l-serine is a protective factor. d-Serine level has a predictive value for HI in uremic patients. Uremic patients are recommended hearing assessment, estimation of d-serine levels, and early intervention.

## Introduction

The aging population has increased the number of patients with chronic kidney disease (CKD) year-by-year, such that the prevalence of the disease in adults worldwide has risen to 14.3% [1], thereby increasing the number of uremia patients and thus the economic burden. CKD patients with disease progression exhibit damaged multiple systems and various complications, including anemia, cardiovascular disease, and cognitive dysfunction. Some studies have shown that the prevalence of sensorineural hearing loss in CKD patients is 36–81%; especially in those with uremia patients, the risk of hearing impairment (HI) is significantly higher than in normal population [2–5]. HI is the fifth leading cause of disability worldwide [6] and a risk factor for cognitive dysfunction in patients [7]. Moreover, HI is independently associated with dementia [8,9] and is prone to anxiety and depression [10,11], affecting the quality of life of patients.

d-Serine is a non-essential amino acid and an enantiomer of l-serine, mainly distributed in the mammalian brain and retina under normal circumstances of low peripheral content. It regulates brain development, neuronal excitability, and synaptic plasticity, and plays a critical role in the central nervous system [12]. Serine racemase (SR) catalyzes the conversion of l-serine to d-serine, while d-amino acid oxidase (DAO) oxidizes and degrades d-serine [13,14]. d-Serine metabolic disorder leads to neurodegenerative diseases and may be involved in mental illness [15]. Some studies have shown that d-serine is nephrotoxic and damages the proximal renal tubules [16]; this feature can be used to evaluate the renal function as well as the early diagnosis and prognosis of CKD [17,18]. However, whether d-serine is related to HI in uremic patients is yet to be clarified. Therefore, the present study aimed to analyze the factors affecting HI and explore the expression of serum d-serine in the peripheral blood of uremic patients with HI and its predictive value. This would provide novel ideas for the clinical prevention and treatment of uremic patients with HI.

## Materials and methods

### Study design and patients

Hearing was measured in uremic patients undergoing maintenance hemodialysis at the hemodialysis center of the First Affiliated Hospital of Kunming Medical University (Kunming, China) from May to August 2022. A total of 30 patients with uremia complicated with HI were enrolled. Also, according to the principle of the case-control study, 30 uremic patients with normal hearing were selected.

All patients underwent hemodialysis 4 h/session, three times a week. Among them, six patients used central venous catheter for dialysis, and the remaining 54 used arteriovenous fistula for dialysis.

These experiments were performed under a project license (no. 2022 EA L 45) granted by Institutional of The Ethics Committee of the First Affiliated Hospital of Kunming Medical University and were conducted in accordance with the Declaration of Helsinki. All participants read and signed the written informed consent form.

Inclusion criteria: patients ≥18-years-old and ≤60-years-old; patients with consciousness and communication ability; glomerular filtration rate (GFR) <15 mL/min·1.73 m^2^; receiving hemodialysis three times/week for >3 months.

Exclusion criteria: patients <18-years-old and >60-years-old; recent major surgery; long-term noise exposure, history of acute otitis media, history of long-time using ototoxic drugs (such as aminoglycoside antibiotics, anti-tumor drugs, analgesic-antipyretic, antimalarial drugs, and loop diuretics for >30 days), family history of deafness; patients with malignant tumors; hemodialysis induction period; unwilling to cooperate.

Some uremic patients used loop diuretics for a short period. A previous study has shown that loop diuretics can cause temporary hearing loss but rarely permanent deafness [19]. Therefore, we excluded patients who were using loop diuretics for >30 days and exhibited HI after subsequently.

## Observation indicators and detection methods

### Questionnaire survey

A structured questionnaire was used to collect information on age, gender, marital status, education level, economic level, comorbidities (hypertension and diabetes), and complications (renal anemia and renal osteodystrophy).

### Physical examination

The height, weight, body mass index (BMI), waist circumference, hip circumference, blood pressure, heart rate, grip strength, and other parameters were measured.

Thin: BMI <18.5 kg/m^2^;

Normal weight: BMI in 18.5–23.9 kg/m^2^;

Overweight: BMI 24.0–27.9 kg/m^2^;

Obesity: BMI ≥28.0 kg/m^2^;

Central obesity: male waist circumference ≥85 cm or waist–hip ratio ≥0.90, female waist circumference ≥80 cm or waist–hip ratio ≥0.85.

### Audiological examination

A Conera pure tone audiometer (MADSEN Co., Ltd., Copenhagen, Denmark) was used to perform pure tone auditory (PTA) in both ears at the frequencies of 250, 500, 1000, 2000, 4000, and 8000 Hz, and the mean values of 500, 1000, 2000, and 4000 Hz were considered as the average hearing thresholds.

First, the medical history was collected, and then the otolaryngologist checked that the external auditory canal and tympanic membrane. According to the above examination, traumatic, sudden, and progressive deafness were excluded. Subsequently, the difference in air conduction and bone conduction in PTA <10 dB was considered sensorineural deafness.

HI criteria: the average hearing threshold ≥25 dB HL was defined as HI.

### Laboratory examination

The laboratory test results of the subjects with respect to hemoglobin (Hb), albumin (ALB), blood urea nitrogen (BUN), creatinine (Cre), uric acid (UA), potassium, calcium, phosphorus, and parathyroid hormone (PTH), were collected.

*Serine level*: The patients receiving dialysis 3 times/week. To avoid the influence of HD on laboratory data, blood was not collected during and one day after HD. A volume of 5 mL venous blood samples was collected from all subjects after 8 h of fasting. The supernatant was collected by centrifugation at *g*-force  =  1167×*g* for 10 min. d-Serine and l-serine levels were detected using DL-Serine Assay (Fluorescent) kits (Abcam Co. Ltd., Shanghai, China, batch no. ab241027). The operation was carried out according to the manufacturer’s instructions.

### Statistical analysis

Databases were established by EpiData 3.1 software and analyzed using SPSS 22.0 (IBM, Armonk, NY). The measurement data were expressed as mean ± standard deviation, and the differences between groups were analyzed using Student’s *t*-test. Chi-square test and Fisher’s exact probability method were used to compare the constituent ratio of the categorical variables. The influencing factors of HI in uremic patients were analyzed by multivariate logistic regression. *α*  =  0.05 (bilateral) was set as the test level, and *p* < .05 indicated a statistically significant difference.

## Results

### Comparison of two groups of patients with basic uremia conditions

Gender, comorbidities (diabetes and hypertension), complications, ALB, potassium, calcium, phosphorus, UA, BMI, and central obesity were evaluated. The results did not find any significant difference in these parameters between the uremic HI and the uremic hearing normal groups (*p*> .05; Table 1).

**Table 1.** Comparison of basic conditions of the two groups of uremia patients.

Item		Normal hearing samples (*n* (%))	HI samples (*n* (%))	*p*
Gender	Male	15 (50.0)	22 (73.3)	.063
	Female	15 (50.0)	8 (26.7)	
Diabetes	No	27 (90.0)	24 (80.0)	.472
	Yes	3 (10.0)	6 (20.0)	
Hypertension	No	0 (0.0)	0 (0.0)	
	Yes	30 (100.0)	30 (100.0)	
Renal anemia	No	12 (40.0)	15 (50.0)	.436
	Yes	18 (60.0)	15 (50.0)	
ALB (40–55 g/L)	≥35	29 (96.7)	27 (90.0)	.612
	<35	1 (3.3)	3 (10.0)	
Potassium (3.5–5.3 mmol/L)	<5.3	15 (50.0)	19 (63.3)	.297
	≥5.3	15 (50.0)	11 (36.7)	
Calcium (2.11–2.52 mmol/L)	<2.52	26 (86.7)	27 (90.0)	1.00
	≥2.52	4 (13.3)	3 (10.0)	
Phosphorus (0.85–1.51 mmol/L)	<1.51	2 (6.7)	4 (13.3)	.671
	≥1.51	28 (93.3)	26 (86.7)	
BMI (kg/m^2^)	Thin	6 (20.0)	3 (10.0)	.637
	Normal	14 (46.7)	18 (60.0)	
	Overweight	7 (23.3)	7 (23.3)	
	Obesity	3 (10.0)	2 (6.7)	
Central obesity	No	15 (50.0)	11 (36.7)	.435
	Yes	15 (50.0)	19 (63.3)	
UA (male: 208–428 μmol/L; female: 155–357 μmol/L)	Normal	6 (20.0)	4 (13.3)	.488
	Raise	24 (80.0)	26 (86.7)	

HI: hearing impairment; ALB: albumin; BMI: body mass index; UA: uric acid.

### Comparison of age, renal function, PTH, and serine levels between the two groups of uremia patients

The comparison of the two groups of uremia patients revealed that age and d-serine level of uremia were higher in the HI group than in the uremia normal hearing group, while the l-serine level was lower than that of the uremia normal hearing group (*p* < .05) and no significant difference was detected in other variables (Table 2).

**Table 2.** Comparison of age, renal function, PTH, and serine levels between the two groups.

Item	Normal hearing (*n* = 30)	HI (*n* = 30)	*t*	*p*
Age (years)	40.73 ± 9.24	46.67 ± 10.93	–2.271	.027
BUN (mmol/L)	28.83 ± 6.58	28.38 ± 7.65	0.246	.807
Cre (μmol/L)	1179.00 ± 289.28	1104.41 ± 290.44	0.997	.323
PTH (pg/mL)	609.71 ± 468.27	634.57 ± 657.44	–0.169	.867
d-Serine (μM)	9.09 ± 2.71	12.27 ± 2.18	–5.003	<.001
l-Serine (μM)	11.81 ± 4.40	9.20 ± 5.33	2.068	.043

BUN: blood urea nitrogen; Cre: creatinine; PTH: parathyroid hormone.

Reference range: BUN 3.6–9.5 mmol/L (male), 2.6–7.5 mmol/L (female); Cre 57–111 μmol/L (male), 41–73 μmol/L (female); PTH: 12–88 pg/mL.

### Influencing factors of HI in uremic patients

HI in patients with uremia was used as the dependent variable. The variables with a significant difference in the measurement and classification data, including age (1 < 43 years, 2 ≥ 43 years), d-serine (1 < 10 μM, 2 ≥ 10 μM), and l-serine (1 < 9.2 μM, 2 ≥ 9.2 μM), were included in the multivariate logistic regression analysis. The results showed that the increased age and d-serine level were the risk factors for HI in uremia patients (odds ratio (OR) = 8.081, 7.482), while the increase in serum l-serine level was a protective factor (OR  =  0.213), as shown in Table 3.

**Table 3.** Multiple logistic regression analysis results of HI in uremia patients.

Item	*B*	S.E.	Wald	*p*	OR	OR (95% CI)
Age	2.089	0.767	7.420	.006	8.081	1.797–36.337
d-Serine	2.013	0.773	6.775	.009	7.482	1.644–34.054
l-Serine	–1.548	0.659	5.517	.019	0.213	0.058–0.774
Constant	–1.485	0.864	2.953	.086	0.227	

HI: hearing impairment.

**Figure 1.**
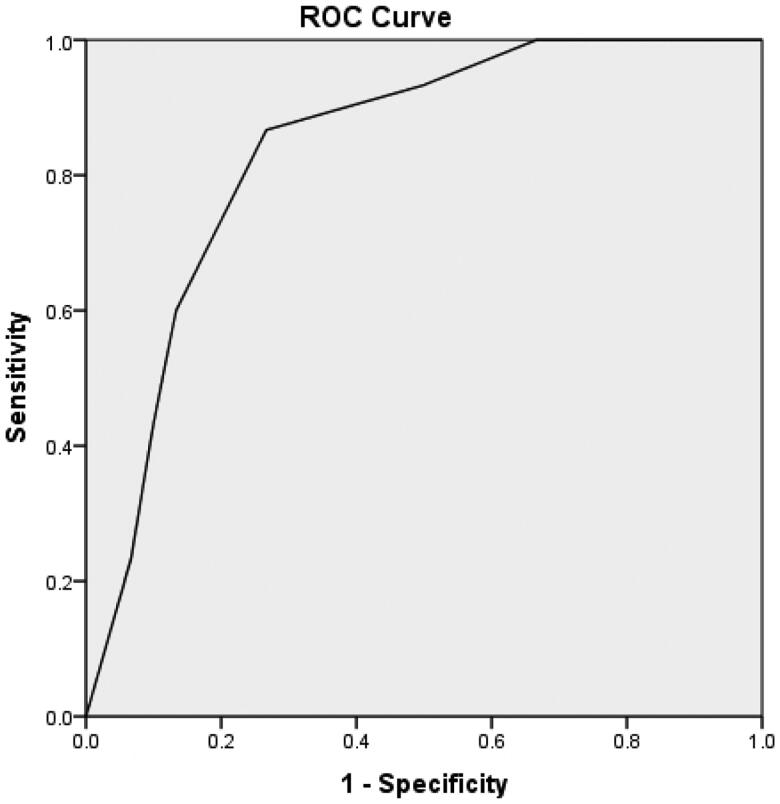
ROC curves of age, d-serine, and l-serine for diagnosing HI in uremic patients.

**Figure 2.**
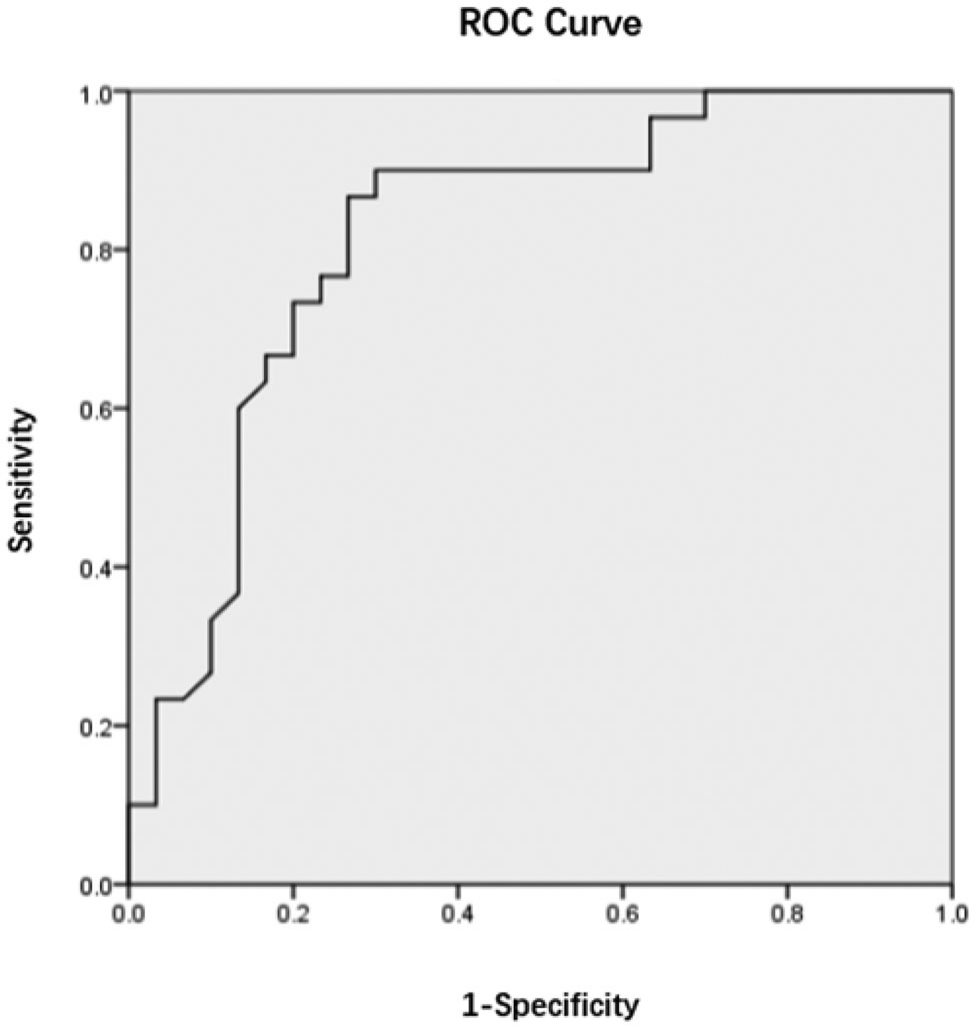
ROC curve of d-serine in the diagnosis of HI in uremic patients.

**Figure 3.**
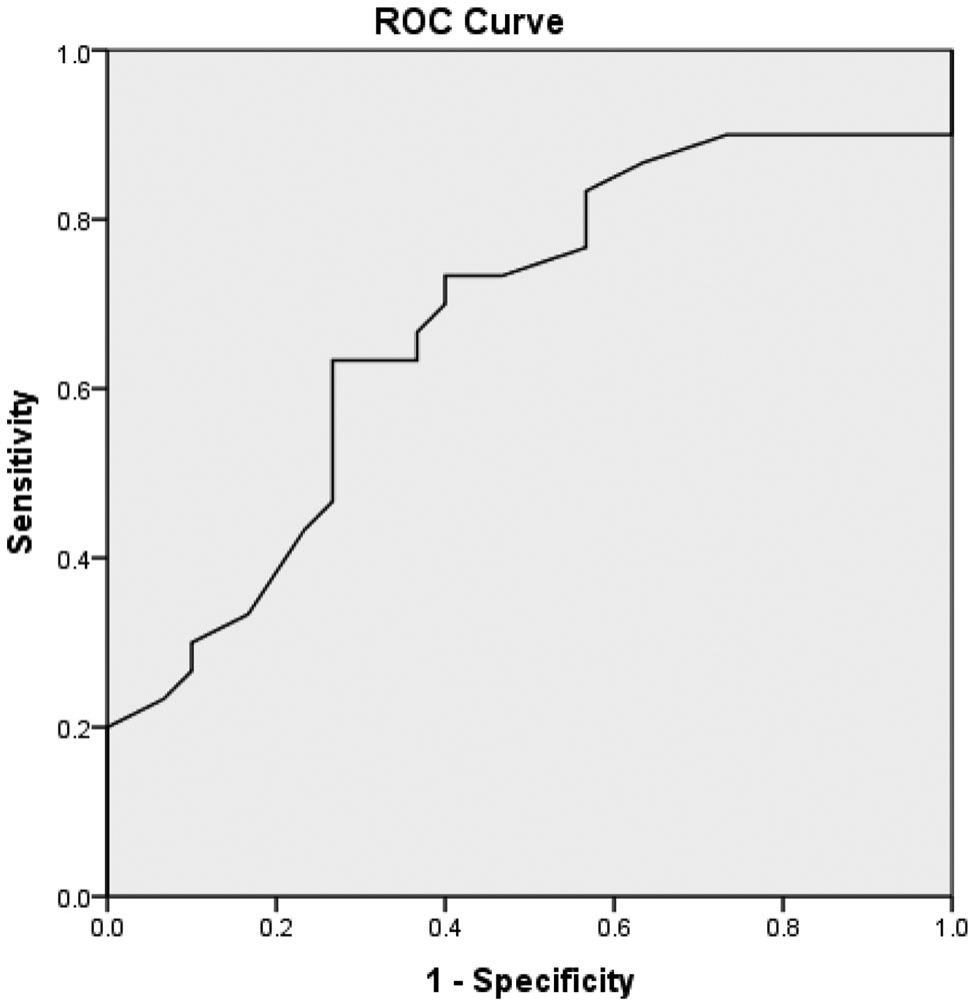
ROC curve of age in the diagnosis of HI in uremic patients.

**Figure 4.**
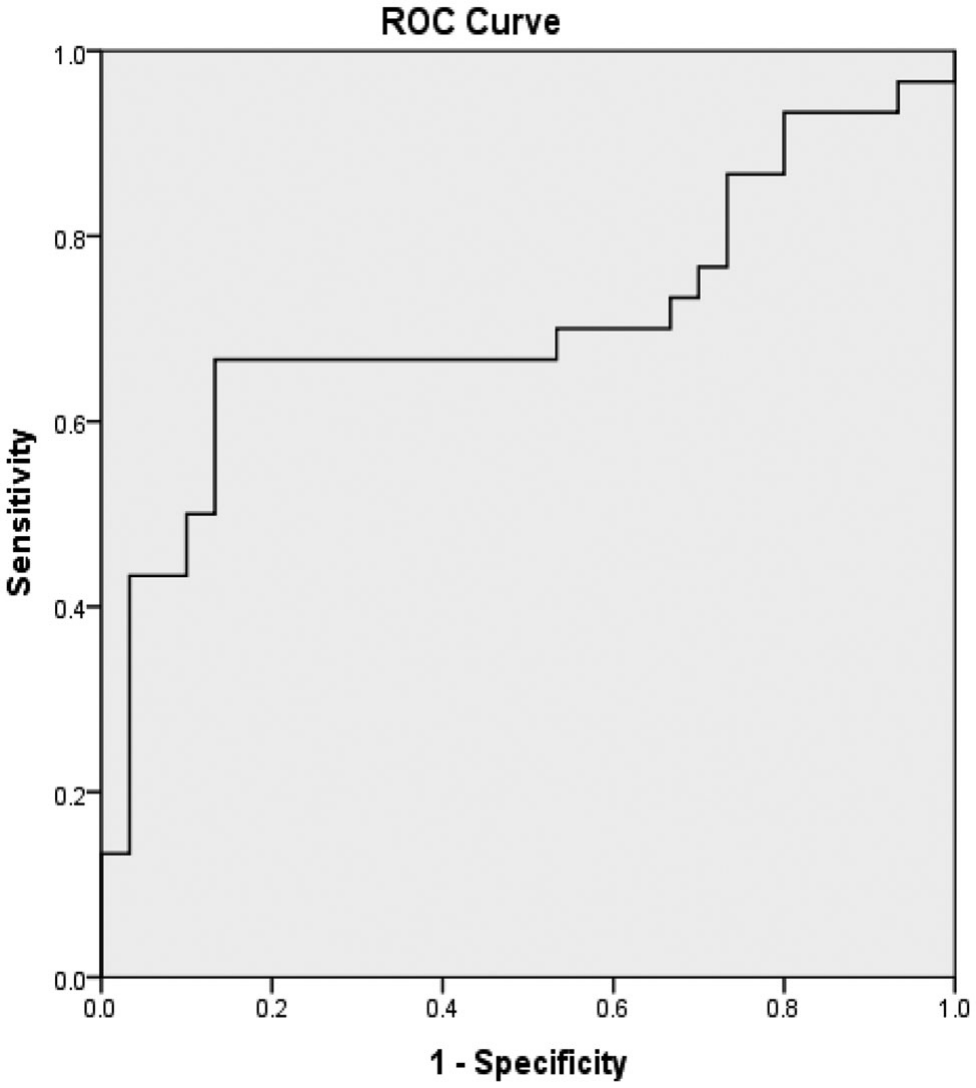
ROC curve of 1/l-serine in the diagnosis of HI in uremic patients.

According to the binary logistic regression analysis, age, d-serine, and l-serine were entered into the model (*p* < .05), and age ≥43 years and d-serine ≥10 μM were risk factors for HI in uremic patients. Furthermore, the risk score of HI in uremic patients was *Y* =  −1.485 + 2.089 × age + 2.013×d-serine − 1.548×l-serine. The receiver operating characteristic (ROC) curve was drawn according to the predicted probability calculated by the risk score, as shown in Figure 1. The area under the curve (AUC) of age, d-serine, and l-serine was 0.838 (95% confidence interval (CI): 0.734–0.943), indicating that age, d-serine, and l-serine had diagnostic and predictive values for HI in uremic patients (*p* < .001). The AUC of d-serine was 0.822 (95% CI: 0.713–0.932), as shown in Figure 2, indicating a highly accurate diagnostic value (*p* < .001). The AUC of age was 0.679 (95% CI: 0.542–0.817), indicating that its diagnostic value had low accuracy, as shown in Figure 3. The AUC of 1/l-serine was 0.707 (95% CI: 0.568–0.846), as shown in Figure 4, indicating its diagnostic value had certain accuracy (*p* = .006). Moreover, the tolerance was >0.1, and the variance inflation factor (VIF) was <10. Therefore, d-serine and l-serine did not have multicollinearity.

## Discussion

The progression of CKD in patients can affect multiple organ functions. Often, HI is a neglected CKD complication. The risk of HI in CKD patients is higher than in normal individuals. The current study also found that the prevalence of HI in uremic patients was 80.5% [5], but the specific mechanism of CKD leading to HI is unclear. A previous study speculated that uremic toxins inhibit the activity of Na^+^,K^+^-ATPase in the central nervous system and affect the auditory nerve function and ion transport in the inner ear [20,21]. In addition, renal hypertension causes vascular sclerosis, and renal anemia causes ischemia and hypoxia; both can cause microcirculation disorder in the inner ear and induce HI. Moreover, hyperlipidemia causes lipid deposition in the inner ear, increases lipid peroxide, leading to inner ear cell damage, decreases hypoproteinemia in plasma colloid osmotic pressure and body fluid extravasation, and increases the inner ear lymph volume and pressure, thereby inducing HI [22,23]. The renal excretion of some drugs is reduced when renal function declines, and accumulation in the body can increase its ototoxicity; the altered electrolyte balance in the inner ear lymph during hemodialysis may also affect hearing [24]. HI is closely related to anxiety, depression, cognitive dysfunction, and dementia [25,26]. If HI is detected in uremic patients, they will suffer from dual diseases and face an increased risk of dementia and depression, decline in quality of life, and poor prognosis [27].

The analysis of the influencing factors of HI in uremia revealed that the risk score of HI was *Y* =  −1.485 + 2.089 × age + 2.013×d-serine − 1.548×l-serine. Hearing decreases with increasing age [28]. The level of d-serine in uremic patients with HI was significantly higher than that in uremic patients with normal hearing. The risk of HI in uremic patients with d-serine ≥10 μM was 5.63 times higher than in patients with d-serine <10 μM. Typically, this result should consider the putative bias factors caused by the small sample size in the study, prompting us to expand the sample size at the later stage. Serum l-serine is a protective factor for hearing, but the specific upstream and downstream mechanisms are yet unclear. Several studies have shown that l-serine can be converted to d-serine under the action of SR, and DAO accelerates the degradation of d-serine. Whether SR is overexpressed or DAO response is reduced in uremic patients, resulting in an increase in d-serine content, is yet to be elucidated.

d-Serine is a rodent renal toxin that causes proximal tubular necrosis [16,29]. *In vitro* experiments have demonstrated that d-serine induces human proximal tubular cell senescence and apoptosis [30]. Thus, it is considered a new type of uremic toxin, and patients’ age, gender, and physique have no significant correlation [17]. Elevated d-serine levels are associated with poor prognosis. CKD in patients with high d-serine levels progresses rapidly, enters the renal replacement therapy stage early, and faces an increased risk of mortality [31]. Some studies have shown that the inner ear and kidney have similarities in anatomical structure, physiological function, immune function, and pharmacological action [32,33]. Cochlear stria vascularis cells and proximal renal tubular cells are rich in mitochondria, have a high oxygen consumption, are vulnerable to hypoxia, and have the same ion exchange function to complete the active transport of liquid and electrolyte, which is crucial for the inner ear to maintain high potassium in the endolymph and the kidney and regulate water and electrolyte metabolism. Furthermore, d-serine has been determined to cause damage to the proximal renal tubules, and there is a similarity between cochlear stria vascularis cells and proximal renal tubular cells. Therefore, it is speculated that d-serine causes damage to cochlear stria vascularis cells and hearing. Some studies have shown that endogenous d-serine is a neurotransmitter in the central nervous system and has a neural regulatory role, including synaptic plasticity, sensory information transmission, neurodevelopment, neurotoxicity, learning, and memory. The present study suggested that the area of the d-serine ROC curve in the uremia with HI group has a high accuracy in the diagnosis of HI. Therefore, it is speculated that the overexpression of d-serine in uremic patients may cause neurotoxicity and ultimately damage hearing. However, the specific mechanism of d-serine on HI is yet unclear. In future studies, we aim to conduct animal experiments to investigate the expression of d-serine, SR, and DAO in the kidney and cochlea under the condition of renal failure combined with HI and analyze the effect of d-serine on hearing and its upstream and downstream correlations.

## Limitations

(1) Small sample size cannot fully reflect the characteristics of HI in overall CKD patients; (2) only d-serine level was detected, and its influencing factors, such as SR and DAO, were not detected and analyzed; (3) this study recruited uremic patients as the research subjects, and hemodialysis treatment may affect the hearing and d-serine levels. In future studies, we intend to expand the sample size to include pre-dialysis CKD patients for comparison, further confirm the predictive value of d-serine level for HI in CKD patients, and explore the mechanism of d-serine on the inner ear.

## Conclusions

Elevated d-serine and age are two risk factors for HI in uremia patients, while l-serine is a protective factor. d-Serine level has a predictive value for HI in uremia patients. Thus, uremia patients should be assessed for hearing in the early stage, and the level of d-serine should be monitored. Early screening of high-risk patients with HI is convenient for early intervention to reduce the disability rates and improve the quality of life and prognosis.
